# Multi-Spectroscopic Characterization of MgO/Nylon (6/6) Polymer: Evaluating the Potential of LIBS and Statistical Methods

**DOI:** 10.3390/polym15153156

**Published:** 2023-07-25

**Authors:** Amir Fayyaz, Haroon Asghar, Muhammad Waqas, Asif Kamal, Wedad A. Al-Onazi, Amal M. Al-Mohaimeed

**Affiliations:** 1 National Centre for Physics, Quaid-i-Azam University Campus, Islamabad 45320, Pakistan; 2 Department of Aerospace and Mechanical Engineering, The University of Arizona, Tucson, AZ 85721, USA; 3 Department of Mining and Geological Engineering, The University of Arizona, Tucson, AZ 85719, USA; 4 Department of Plant Sciences, Faculty of Bioscience Biological Sciences, Quaid-i-Azam University, Islamabad 45320, Pakistan; 5 Department of Chemistry, College of Science, King Saud University, P.O. Box 22452, Riyadh 11495, Saudi Arabia

**Keywords:** polymer, PCA, laser-induced breakdown spectroscopy, EDX, XPS, FTIR, XRD, ICP-MS

## Abstract

The potential of using laser-induced breakdown spectroscopy (LIBS) in combination with various other spectroscopic and statistical methods was assessed for characterizing pure and MgO-doped nylon (6/6) organic polymer samples. The pure samples, obtained through a polycondensation chemical technique, were artificially doped with MgO prior to analysis for comparative purposes. These artificially doped samples served as crucial reference materials for comparative analysis and reference purposes. The LIBS studies were performed under local thermodynamic equilibrium (LTE) and optically thin plasma conditions. To assess the structural crystallinity of the nylon (6/6) polymer samples, X-ray diffraction (XRD) analysis, and Fourier transform infrared (FTIR) spectroscopy were employed to detect functional groups such as N-H, C-H, and C-N in the adsorbent polyamide nylon sample. Additionally, diffuse reflectance spectroscopy (DRS) analysis was conducted to investigate the effects of doping and temperature on the band gap and material reflectance across different sample temperatures. Chemical compositional analysis was performed using X-ray photoelectron spectroscopy (XPS) with the carbon C1s peak at 248.8 eV serving as a reference for spectrum calibration, along with energy-dispersive X-ray (EDX) analysis, which demonstrated good agreement between the techniques. To validate the different methodologies, the results obtained from CF-LIBS and EDX were compared with those from the standard inductively coupled plasma mass spectrometry (ICP-MS) technique. Finally, for classification analysis, principal component analysis (PCA) was applied to the LIBS spectral data at different sample temperatures (25 °C, 125 °C, 225 °C, and 325 °C). The analyses demonstrated that the combination of LIBS with PCA, along with other methods, presents a robust technique for polymer characterization.

## 1. Introduction

Laser-induced breakdown spectroscopy (LIBS) is a highly versatile analytical technique that is capable of performing qualitative and quantitative chemical analysis on a wide range of materials. It has been successfully applied in numerous fields, including but not limited to, plastics [1], alloys [2], rocks [3,4], aerosols [5], cement powders [6], radioactive substances [7], fissionable materials [8], explosives [9], polymers [10,11], mineral exploration [12], mining [13,14,15,16,17], and waste industrial substances [18,19,20,21]. LIBS offers the ability to characterize and measure the elemental composition and concentration of these materials, thereby making it an invaluable tool for various research, industrial, and environmental applications. LIBS optical emission signals from the laser-stimulated plasma (LSP) are used to find the ingredient elements, as well as their composition in the sample under assessment. In the LIBS literature, most of the research studies focused on the neutral and ionic emission spectra. However, molecular emission spectra from the LSP contain more advanced chemical evidence that can be developed to investigate the materials. LIBS has been exploited as a real-time, quick, stand-off, non-damaging, and in situ detection spectroscopical technique for the study of different organic, mineral, and explosive textiles [22]. LIBS can also be combined with other spectroscopic methods such as Fourier transform infrared (FTIR), energy dispersive X-ray (EDX), X-ray photoelectron spectroscopy (XPS), X-ray diffraction (XRD), and diffuse reflectance spectroscopy (DRS) to improve the capacities of LIBS for elemental analysis and to gain a better structural understanding of organic polymers.

Polymeric fabrics containing nano-fibers, nano-wires, and nano-rods are growing in their attractiveness for polymer researchers and manufacturers owing to their efficient mechanical and physical properties; for instance, they provide strength, spinnability, flexibility, and luster in textile products, among others [23,24]. Polymeric materials coupled with nano-metal oxides can enhance the functional characteristics of textile materials to confer durability, thermal resistance, UV safeguarding, self-scrubbing, spark retardancy, and anti-infectious qualities. Polyamide materials are fabricated through numerous market labels. For example, nylon (6/6) and nylon 6 are some examples. Polyamide materials can be classified into aliphatic and aromatic classes. The word polyamide is the principal classification of polymers based on the engineering thermoplastic resin used in the polymer industry and has extremely remarkable material features. Nylon (6/6) organic polymer is one of the exclusive polymeric fabric substances commonly that is used in scientific research and textile industries due to its high thermal strength, hydro-phylicity, and extremely flexible power [25]. Magnesium oxide (MgO) nanoparticles embedded into the nylon polymeric matrix have great sensible applications regarding the mechanical power and strength of the non-woven materials. The polyamide materials based on doped nylon have been widely explored for years in various industrial fields, including motorized, wrapping, electronic and integrated circuit technology, and athletic and lubricant and gas productions, due to their exclusive blends of topographies, such as an elevated melting point, low chemical responsiveness, robustness, strength, high-temperature properties, and informal processing [26]. In recent years, the requirements of polyamide nylon products compared to metallic materials has enhanced extensively in motorized and energy train industry system. The chemical structure of polyamide nylon polymers depends on amide ensembles that contribute to bonding and enhances the range of the melting temperature. However, from a structural and chemical quantification point of view, there is still a lack of research on doped polyamide nylon polymer materials for to their thermal and mechanical characteristics.

Pistiki et al. [27] investigated various polymers as calibration agents for deep-UVRR and estimated their function in separating clinically isolated bacterial genera. Jose Carlo et al. [28] studied a polymeric film for identifying ionic zinc, Zn (II) in aqueous media using photographs, and the results have been contrasted with inductively coupled plasma mass spectrometry (ICP-MS). Boueri et al. [29] described the experimental setup for eight polymer samples, which discussed the optimization of an artificial neural network (ANN) model. They included the learning and validation phases using the same eight polymer samples. In addition, they enhanced the performance of the ANN regarding ten PVC samples and detected minor or trace metallic elements to characterize sub-categories based on their concentrations. Farooq et al. [30] evaluated the elemental analysis of polystyrene and polycarbonate used in food cans by employing laser-induced breakdown spectroscopy. Trautner et al. [31] reported the identification and replication of molecular C_2_ Swan and CN violet and red bands in standard polymer samples. Janjuri et al. [32] investigated various kinds of post-consumer plastics by using the laser-induced breakdown spectroscopy technique. Costa et al. [33] described an alternate procedure for the detection and categorization of six different kinds of e-waste polymers. Banaee et al. [34] studied the classification of recyclable polymers using discriminant function analysis (DFA) coupled with LIBS data. Recently, Sommer et al. [35] studied the effect on the sample’s surface and its depth profile for the weathering-induced oxidation of polystyrene target samples at different weathering times using the LIBS methodical tool. More recently, Nie et al. [36] analyzed six various colored plastics such as POM, PP, ABS, PVC, PE, and PA using LIBS blended with (NCA) and support vector machine (SVM) statistical analysis. Therefore, the identification, classification, and characterization of polymers used in industries based on polymeric materials in different atmospheres and temperatures are significant. For various grounds, nylon (6/6) polyamide polymer material is a potential substance that is also used to improve the chemical properties of cement powder and Li-ion batteries [37,38].

The utilization of the principal component analysis (PCA) method in the context of LIBS data is indispensable in today’s industrial setting for the examination of nylon Polymer (6/6) and doped nylon (6/6) materials. This integrated approach allows for a holistic comprehension of the material’s elemental composition, establishes correlations between the composition and properties, facilitates rapid analysis, and enables effective process monitoring. By harnessing the capabilities of PCA-LIBS analysis, industries are empowered to enhance material quality, optimize performance, and foster innovation across diverse sectors such as textiles, automotive, aerospace, and electronics. Despite the significance of comprehensively assessing the optical characteristics of nylon (6/6) polymer material, there has been a dearth of methodical investigations in this area. This study combines LIBS, statistical analysis, and optical techniques to investigate doped nylon polymers, thereby aiming to understand their elemental compositions and optical properties. It addresses a gap in the literature by comprehensively analyzing the optical characteristics of nylon (6/6) polymer material using LIBS and statistical analysis. The study also introduces the novel application of LIBS for analyzing the optical properties of doped polymers, thereby presenting a valuable methodology for future polymer characterization research. Overall, this research advances the field by providing insights, methodologies, and potential directions for further investigations into polymer analysis.

In the initial phase, a thorough analysis using LIBS was conducted, thus ensuring the presence of local thermodynamic equilibrium (LTE) and an optically thin plasma condition. In the second phase, structural analysis and estimation of the crystallinity in the nylon (6/6) polymer material were carried out using XRD analysis. The third phase involved SEM-EDX analysis to examine the surface morphologies and elemental composition of the polymer sample. In the fourth phase, FTIR spectroscopy was employed to identify functional groups such as N-H, C-H, and C-N in the adsorbent polyamide nylon sample. The fifth phase utilized XPS to analyze the chemical composition of elements on the surface of the same nylon polymer sample, with the carbon C1s peak at 248.8 eV used as a calibration reference. In the sixth phase, DRS analysis was performed to determine the reflectance as a function of the wavelength at different sample temperatures. Additionally, the bandgap energies of pure and MgO-doped nylon (6/6) polymer samples were estimated. Finally, for classification analysis, PCA was conducted on the LIBS spectral data acquired at different sample temperatures, including 25 °C, 125 °C, 225 °C, and 325 °C.

## 2. Materials and Methods

### 2.1. Experimental Setup

**Figure 1:**
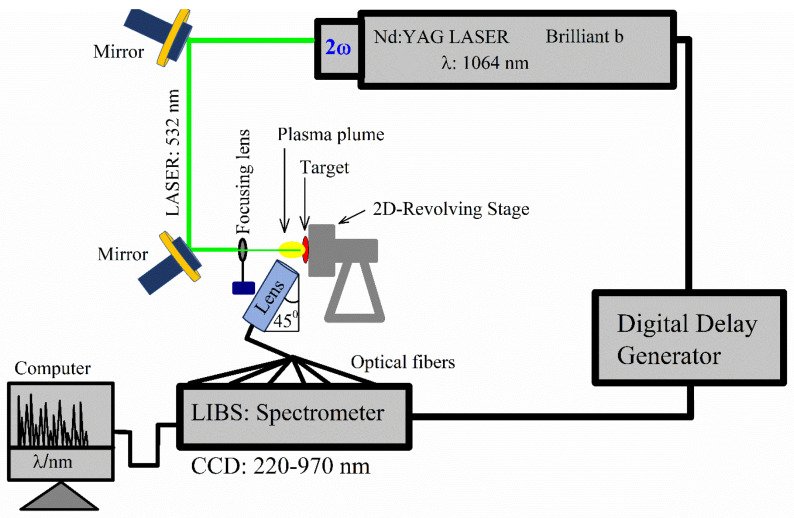
LIBS experimental setup used for the study of MgO-doped nylon (6/6) polymer.

Figure 1 illustrates the graphic diagram depicting the experimental setup employed in this study for LIBS. The experimental setup involved the utilization of a Q-switched Nd: YAG laser, which generated a high-power laser beam with specific parameters—2nd harmonic wavelength of 532 nm, maximum laser pulse energy of 400 mJ, pulse duration of 5 ns, and a repetition rate of 10 Hz. The flash lamp voltage was adjusted to achieve a laser pulse energy of 100 ± 0.63 mJ, thereby ensuring a good signal-to-noise ratio and avoiding signal saturation. The laser beam was directed through two mirrors and then focused onto the target with a shallow focus using a quartz lens having a focal length of 30 cm. This focusing process was responsible for generating plasma. Subsequently, the plasma’s optical spectrum was collected using optical fibers (multi-mode) with a collimating lens having a high OH and core diameter of ~600 μm. The spectrometer (Avantes, Apeldoorn, The Netherlands) used had an integration time of 10 μs and covered a wavelength range of 220–970 nm to record the optical emission spectrum. The instrumental resolution of our system was (0.06 ± 0.01) nm at 600 nm. At a wavelength of 600 nm, the instrumental resolution of our system measured (0.06 ± 0.01) nm. To ensure variation in the ablation spots of the laser beam, the sample was positioned on a two-dimensional stage that rotated at a speed of 12 rpm. In order to achieve a high and unsaturated spectral signal with a good signal-to-noise ratio (SNR), a time delay of 2.5 μs was set between the triggering of the laser pulse and the opening of the detection window. The laser beam spot size, estimated to be approximately 78 μm, had a diffraction-limited diameter. Furthermore, the LIBS experiment was conducted in an atmospheric environment, specifically, in the air.

To study the crystal structure of the nylon (6/6) organic polymer sample, the XRD analysis was achieved by using the Bruker D8 Advance X-ray diffractometer with Cu-K_α_ radiation having a wavelength of λ = 0.1542 nm operated at 40 kV and a beam current of 30 mA. The scanning speed, dwell time, and step size were 0.3 step size/s, 12 s per increment, and 0.05°, respectively. EDX is an analytical technique employed to detect and describe the chemical concentration of the sample. For the EDX analysis, an Oxford Instrument (X-MAXN-20 EDX) connected to a scanning electron microscope (SEM) having a depth profile of about 2 microns operated at 20 keV was used to investigate the chemical composition of the nylon (6/6) polymer sample. The X-rays emitted from the target polymer sample were recorded using a Si (Li) detector having a size of ~30 mm^2^.

The FTIR technique was employed to interpret the raw data (interferogram) into the infrared spectrum of transmission or absorption as a function of wavenumber (cm^−1^) through the mathematical procedure. FTIR analysis detects the existence of carbon-based organic and inorganic complexes in a sample. The functional groups that occurred in the nylon (6/6) polymer sample were investigated using a NICOLET-6700 FTIR spectrometer. The FTIR spectral data was registered in the range from (4 × 10^3^–4 × 10^2^) cm^−1^ at a high-depth resolution of 4 cm^−1^. The XPS technique with an excitation X-ray source of Al-Kα at 1486.9 eV under an ultra-high vacuum of ~10^−9^ Torr was applied to analyze the nylon polymer sample. The XPS experiment was performed under a high vacuum to avoid the absorption of photoelectrons in the air particles. The optical properties of the prepared nylon (6/6) sample were studied through a DRS (PerkinElmer LAMBDA-950 UV–VIS–NIR spectrometer) having a wavelength ranging from 200–1200 nm. For the PCA analysis, MATLAB R2020b was employed for data pre-processing, and then the ioGAS™ software program was used to achieve the classification study. The PCA analysis was applied using a set of 18 spectral lines comprising both atomic (I) and singly ionized (II) emission lines. These lines consist of H (I) at 434.0 nm, 486.1 nm, and 656.3 nm; C (I) at 247.8 nm, 906.1 nm, and 940.5 nm; Na (I) at 588.99 nm, 589.59 nm, and 819.5 nm; Mg (II) at 279.5 nm, 280.3 nm, and 285.2 (I) nm; Al (I) at 309.3 nm, 394.4 nm, and 396.2 nm; and Si (I) at 250.6 nm, 251.6 nm, and 288.2 nm. Inductively coupled plasma mass spectrometry (ICP-MS) is an elemental analysis analytical technology capable of analyzing the composition of a sample at a trace level as low as less than 1 ppm. In ICP-MS analysis, the sample is chemically ionized by the thermal plasma and then the mass analyzer separates ions based on their mass-to-charge ratio using the electric and magnetic fields. The plasma is generated at a 1.6 kW power, and Ar gas was also utilized for supplemental and cross-flows at a 10 L/min flow rate.

### 2.2. Sample Preparation

The organic polymer material known as nylon (6/6) consists of two monomers: hexamethylene diamine and adipic acid. Its chemical formula is [(C_12_H_22_N_2_O_2_)]. The valence shell electronic configurations for the elements C (carbon), H (hydrogen), N (nitrogen), and O (oxygen) are as follows: C (2s^2^ 2p^2^), H (1s^1^), N (2s^2^ 2p^3^), and O (2s^2^ 2p^4^). Its lattice assembly is made inside an orthorhombic unit cell with lattice parameters (axial lengths) such as c~0.530 nm, b~0.864 nm, and a~1.725 nm, and the angles are designed as α = β = γ = 90°. The polymerization of a nylon (6/6) sample was achieved by chemically condensing hexamethylenediamine and adipic acid through a process called polycondensation. Equal concentrations of adipic acid and hexamethylenediamine were combined in a vessel along with water (H_2_O). The resulting mixture formed an ammonium–carboxylate salt, which was preserved to obtain nylon salt. Subsequently, the polymerization process occurred continuously within a reaction vessel, which follows the given reaction:

(1) $$n\left[HOOC-{\left(CH_{2}\right)}_{4}-COOH\right]+n\left[H_{2}N-{\left(CH_{2}\right)}_{6}-{NH}_{2}\right]\to {\left[-OC-{\left(CH_{2}\right)}_{4}-CO-NH-{\left(CH_{2}\right)}_{6}-NH-\right]}_{n}+\;(2n-1)H_{2}O.$$

To eliminate moisture from the mixture (consisting of acid and amine functions) and facilitate the formation of polymer amide bonds through the polymerization process, the prepared sediments were subjected to low-temperature drying in an oven. The drying temperature ranged from 20–70 °C, thus resulting in the formation of a chain-like molecular network. To preserve the thermal imaging and crystallinity of the synthetic polymer, the samples were dried at a low temperature not exceeding 70 °C. This temperature range was chosen because higher temperatures tend to decrease the crystallization rate. Following the drying process, a nylon (6/6) film was prepared using polymerization techniques. For the doping procedure, nylon (6/6) was dispersed in formic acid and stirred. A known amount of MgO (15 wt.%) was then added to the nylon (6/6) solution. The incorporation of MgO into nylon (6/6) is highly desirable due to its advantageous thermal conductivity, heat resistance, and anti-microbial properties. MgO particles with a size range of 60–70 nm and a surface area of approximately 40 m^2^/g were mixed using the solution blending method to create a MgO (15 wt.%)/nylon (6/6) solution. The resulting solution mixture underwent sonication (at room temperature) using a sonicator for 40 min to ensure proper dispersion of MgO within the polymer solution. Nano-composite sheets of the MgO/nylon (6/6) sample were prepared according to the aforementioned method. To eliminate any attached chemical particles, the prepared composite film was immersed in sterilized water. Finally, the MgO/nylon (6/6) composites were dried in an oven at 70 °C. To conduct ICP-MS analysis, a sample solution was created by blending 70 mg/kg of finely dehydrated powder of the MgO (15 wt.%)/nylon (6/6) sample with 3 mL of laboratory-grade nitric acid (HNO_3_) and 6 mL of hydrofluoric acid (HF).

## 3. Results and Discussion

### 3.1. LIBS Spectra Analysis

**Figure 2:**
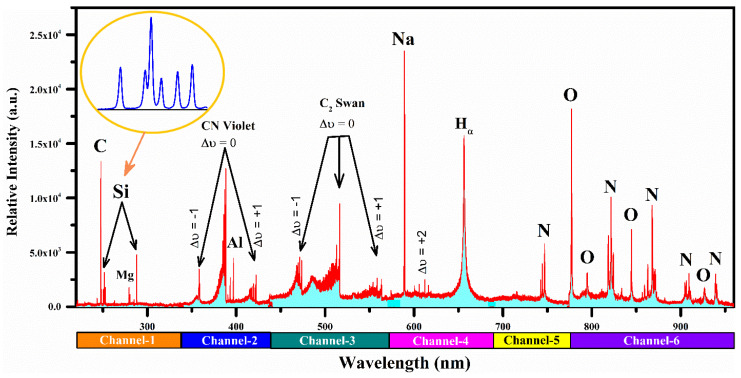
Broadband optical emission spectrum as a function of wavelength generated through the laser-induced plasma of the MgO-doped nylon (6/6) polymer material.

The optimized conditions for recording the emission spectra of the MgO-doped nylon (6/6) polymer material involved various parameters. These included a laser pulse energy of 100 mJ, a beam power of 0.02 GW, a focal length of the focusing lens set at 30 cm, and a distance of 2–3 mm between the plasma plume and the signal collecting lens. The signal-collecting lens was further positioned at a 45° angle relative to the plasma plume. The LIBS emission spectrum was registered using a 6-channel, linear array compact broadband CCD spectrometer. The spectrum was investigated by detecting the spectral lines using the NIST spectral database [39]. The spectral lines of various ingredient elements with different line intensities were detected in the emission spectrum of the MgO-doped nylon (6/6) polymer material sample. Figure 2 represents an averaged spectrum, rather than a single shot spectrum covering the wavelength range from 220–970 nm. We conducted a thorough optimization process to determine the optimal SNR for different numbers of averages. By systematically varying the number of averages, we carefully balanced the SNR to achieve the best possible results. The emission lines correspond to carbon (C), hydrogen (H), sodium (Na), silicon (Si), aluminum (Al), nitrogen (N), and oxygen (O) along with a multiplet bunch of singly ionized magnesium (Mg II), due to the 3p ^2^P_1/2,3/2_ → 3s ^2^S_1/2_ and 3d ^2^D_3/2,5/2_ → 3p ^2^P_1/2,3/2_ transitions. However, it is worth mentioning that numerous structures of Si emission lines at 250.7, 251.4, 251.6, 251.9, 252.4, 252.8, 263.1, 288.1, and 390.6 nm with adequately high spectral line intensities were detected in the sample (see as inset Figure 2). Furthermore, the molecular emissions of the CN violet band due to the transitions from the B^2^Σ^+^ to the X^2^Σ^+^ and the C^2^ Swan band due to d^3^ Π_g_–a^3^ Π_u_ transitions were observed in the wavelength range from 350–425 nm and 450–645 nm, respectively. The spectral emission lines of the CN molecular band were identified at 388.3, 387.1, 386.2, 385.5, and 385.1 nm that were associated with the vibrational transitions (0,0), (1,1), (2,2), (3,3), and (4,4), respectively. The spectral emission lines of the C^2^ Swan band were identified at 467.3, 468.3, 469.8, 471.6, 473.7, 509.6, 512.9, 516.5, 550.2, 554.1, 558.5, 563.5, 600.5, 606.0, and 612.0 nm that were associated with the vibrational transitions (5,4), (4,3), (3,2), (2,1), (1,0), (2,2), (1,1), (0,0), (3,4), (2,3), (1,2), (0,1), (3,5), (2,4), and (1,3), respectively. The transitions among two electronic levels were established using the variance in vibrational quantum statistics from the higher to lower electronic state.

The presence of CN and C_2_ bands in the LIBS analysis of the nylon polymer samples provides valuable information about their molecular compositions and characteristics. These bands reveal insights into the chemical bonding and molecular structure of the polymer. Analyzing the intensity, shape, and position of these bands helps determine properties such as the polymerization degree, impurities, additives, and overall chemical composition. Moreover, the CN and C_2_ bands enable the study of thermal degradations and alterations in the molecular structure due to heat or chemical reactions. Thus, CN and C_2_ bands in LIBS spectral analysis are a powerful tool for extracting information on nylon polymers’ molecular properties, compositions, and behaviors.

The presence of Al and Si in the MgO-doped nylon (6/6) polymer sample can be attributed to several factors. These elements could have originated from impurities present in the raw materials used during the sample synthesis. During the LIBS analysis, even minor or trace amounts of Al and Si can generate detectable spectral lines due to the sensitivity of the technique. The presence of these elements, albeit in low concentrations, can still provide valuable information about the material’s composition and purity. It is worth noting that the detection of Al and Si as minor or trace components in the pure nylon polymer sample may have implications for certain applications where the absence of these elements is critical. Therefore, further investigation into the source and potential mitigation strategies for these impurities would be beneficial for optimizing the purity and quality of the nylon polymer.

For the plasma characterization, the hydrogen (H_α_) emission line profile, which is free from the self-absorption effect at 656.28 nm due to 3d ^2^D_5/2_ → 2p ^2^P_3/2_ transition, was utilized to estimate the plasma electron number density. The PCA of the MgO-doped nylon sample at different temperatures was performed with a set of six selected elements to identify the polymer target demeanor as a function of the temperature. To study the PCA, optically thick and well-isolated atomic spectral lines of several elements such as H (I) at 656.28 nm, C (I) at 247.86 nm, Al (I) at 394.4 nm, Si (I) at 251.61 nm, Na (I) at 588.99 nm, and Mg (I) at 285.21 nm were selected.

Moreover, in this particular study involving LIBS conducted within an air environment, the signal that indicates the presence of H, N, and O can emerge from various sources, which encompass the sample’s composition regarding contamination originating from the surrounding atmosphere and compel the necessity of performing background subtraction. It is imperative to ensure the accurate identification and quantification of the H, N, and O in LIBS experiments by diligently carrying out calibration, utilizing reference materials, and validating the findings through complementary techniques. These essential steps guarantee the reliability and validity of the obtained results, thereby facilitating the interpretation of the samples’ elemental compositions.

### 3.2. Plasma Characterization

**Figure 3:**
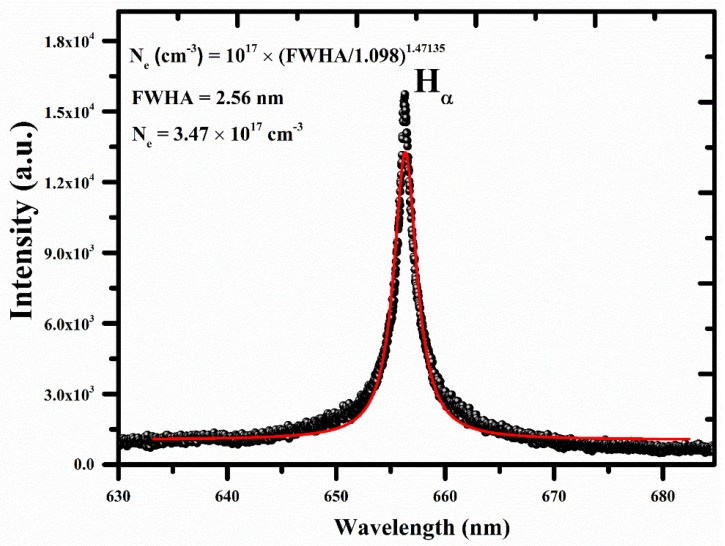
The electron number density of the LTE plasma using line profile of H_α_ emission at 656.28 nm, along with the Lorentzian fit (red color).

In order to characterize the LIBS plasma accurately, certain requirements need to be met, such as the plasma is optically thin and satisfying local thermodynamic equilibrium (LTE) conditions. An optically thin plasma ensures that self-absorption and saturation-free emission line profiles of the atoms are generated, thereby resulting in minimal uncertainties in the electron plasma density and excitation temperature. To confirm the optically thin nature of the plasma, we employed the line intensity comparison technique, as described in reference [40]. The experimentally observed Si I spectral line intensities were compared to the estimated ratio derived from spectroscopic components such as the transition probabilities, statistical weights, and wavelengths. The comparison revealed an excellent agreement within a relative error of 10%, thus strongly indicating that the LIBS plasma was optically thin. In addition, we verified the optically thin plasma condition based on the McWhirter criterion [41]. Therefore, the optical information obtained from the plasma could be reliably used for further characterization purposes. The electron number density was calculated using a Lorentzian line profile of the hydrogen H_α_ emission line at a wavelength of 656.28 nm using Equation (2) [42], as shown in Figure 3. The full width at half maximum (FWHA) of the spectral line was estimated utilizing a Lorentz fitting (red color), which integrated the instrumental width (0.06 ± 0.01 nm). The recorded H_α_ spectral line of the target sample was well-isolated and had a good SNR. The studied Lorentzian FWHA of the H_α_ spectral line was determined to be Δλ_FWHA_ = (2.56 ± 0.3) nm. The estimated Doppler width (ω_D_) employing the excitation temperature was ~0.005 nm, and it was insignificant compared to the Lorentzian width (ω_L_). The estimated plasma electron density from Equation (2) was (3.47 ± 0.3) × 10^17^ cm^−3^.

(2) $$N_{e}={\left(\frac{{∆\lambda }_{FWHA}}{1.098}\right)}^{1.47135}\times 10^{17}{cm}^{-3}$$

**Figure 4:**
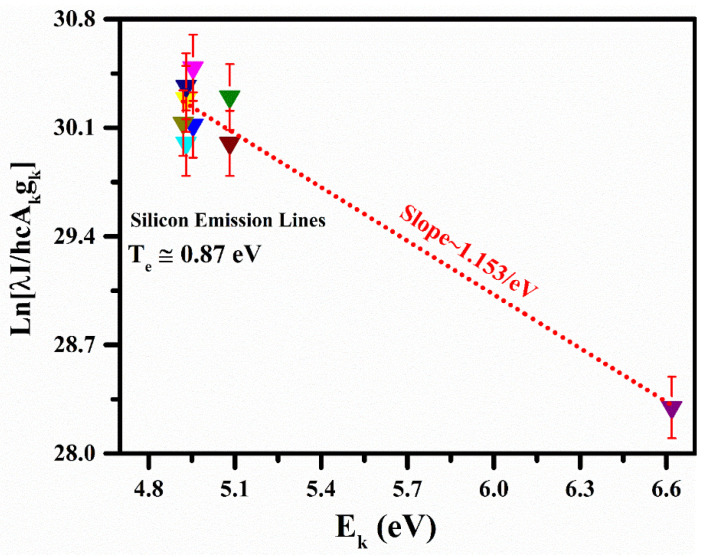
Boltzmann plots were constructed to estimate the plasma excitation temperature.

The plasma temperature was determined from the atomic emission lines of the neutral silicon (Si I) for the MgO-doped nylon polymer sample using the Boltzmann equation [39,40]. The spectroscopic data related to the Si emission lines used to construct the Boltzmann plots are shown in Table 1. The spectroscopical atomic parameters, including the transition probability (s^−1^), upper-level energy (cm^−1^), statistical weight (g_k_), and elemental wavelength (nm), were taken from the NIST atomic database [39]. Usually, inaccuracies in the plasma temperature using the Boltzmann plot technique appear owing to errors in the estimated line intensities and absolute transition probabilities. In this work, the estimated plasma temperature comprised a ~5% relative error. The self-absorption effect in the LIBS study reduces the intensity of a spectral line, and line broadening due to the radiated light absorption of an atom in another portion of the plasma causes an inhomogeneity in the plasma that creates numerous erratic tumbles on the optical emission spectrum. Therefore, self-absorption in the line opens uncertainty in determining the chemical composition using CF-LIBS. In this analysis, we rectified the chosen spectral line intensities for the CF-LIBS to reduce the self-absorption effect, which was less than 5% [43]. In Figure 4, the Boltzmann plot of the polymer sample is presented using the self-absorption rectified Si (I) spectral lines. The slopes (1/k_B_T) of the linear fit to the experimental data points in the Boltzmann plots provide the plasma temperature. The plasma temperature determined from the Si (I) spectral lines was (0.87 eV ± 5%), and the corresponding calculated slope was (1.153/eV). The error bars show the estimated error that was ascribed to the inaccuracies present in the integrated line intensities, as well as the absolute transition probabilities. The estimated uncertainty in the calculated plasma temperature was ~5%.

### 3.3. XRD Analysis

**Figure 5:**
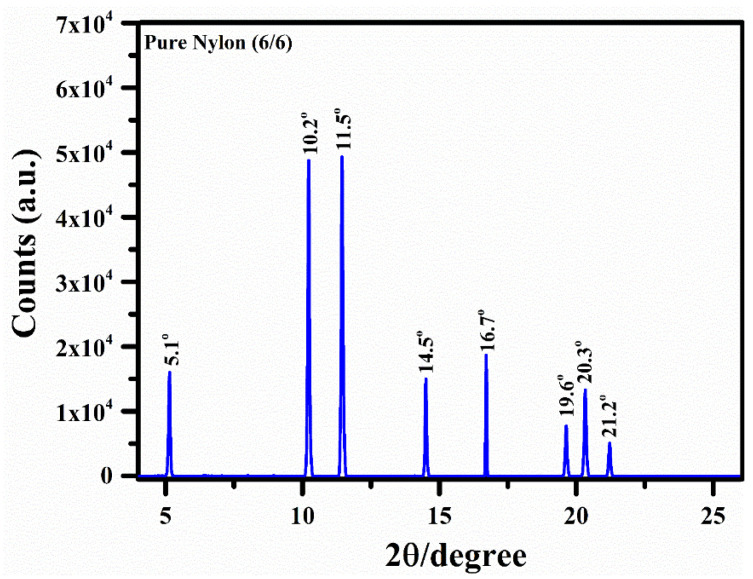
XRD spectrum of nylon (6/6) as a function of diffraction angle (2θ).

Figure 5 shows the XRD spectrum (scanned over 2θ values from 1° to 50°) of the nylon (6/6) as a function of the diffraction (glancing) angle (θ). However, the XRD spectrum for the 2θ values from 4° to 26° is demonstrated in this study. The well-resolved isolated high-intensity peaks can be a periodic layout of atoms, as detected characteristic X-rays are detected in explicit directions. The particular types of characteristic X-ray lines in the spectrum show the level of crystallinity of various materials such as crystalline, semi-crystalline, and amorphous polymers. In addition, the crystalline size of the polymer particles can be estimated using the Debye–Scherrer equation [44].

(3) $$L=\frac{K\lambda }{\beta \cos\theta }$$

Here, *λ* is the wavelength of the incident X-rays, *θ* is the Bragg’s angle, *L* is the crystalline size (nm), *K* is a constant known shape factor with a value of 0.94, and *β* is the full width at the half maximum (FWHM) of the peak. In this study, we did estimate the average crystalline size of the nylon (6/6) polymer sample, and the obtained value was approximately 31 nm. The diffracted X-ray line profiles of the nylon (6/6) can be observed below at various diffraction angles (2*θ*), such as 5.1°, 10.2°, 11.5°, 14.5°, 16.7°, 19.6°, 20.3°, and 21.2°.

### 3.4. SEM-EDX Chemical Analysis

**Figure 6:**
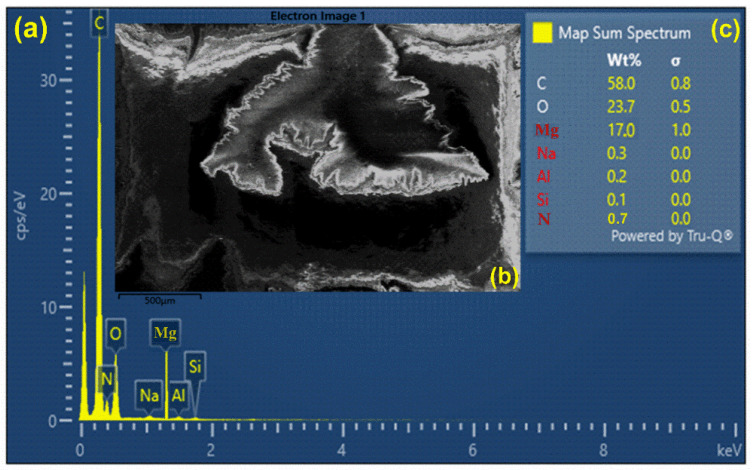
(**a**) EDX spectrum of MgO-doped nylon (6/6) polymer. (**b**) SEM micro-image of the sample. (**c**) Relative wt.% of the elements present in the sample.

The EDX attached with scanning electron microscopy (SEM) is a surface analytical technique having a depth profile of around 5 microns and a detection limit of ~0.1 wt.% for the elemental analysis. The EDX is based on the characteristic X-ray emissions from the excited atoms of the target that confirm the presence of chemical elements in the target sample [45,46]. EDX chemical analysis has already been employed in several fields such as agrochemicals, plant sciences, mineralogy, pharmaceuticals, and metal industries. EDX emission analysis includes qualitative, as well as quantitative, information about the target sample. The EDX spectrum of our nylon (6/6) sample is shown in Figure 6a, which displays the characteristic X-ray emissions due to the K-shell and L-shell electronic transitions. The EDX analysis shows that C, O, and Mg were detected as major ingredient elements, along with Na, Al, Si, and N as trace elements in the nylon (6/6) polymer sample. The SEM test was performed to study the surface morphologies of the nylon (6/6) polymer sample film. Figure 6b shows the SEM image of the pure nylon (6/6) polymer sample thin film at room temperature. The micro-image revealed that nylon (6/6) nanofiber-like thin threads were present at the surface that were <10 nm in diameter and were separated with a bulge boundary. For the compositional analysis, the strongly detected Kα_1_, Kα_2_, and Kβ_1_ peaks of various elements such as C, O, N, Mg, Na, and Si at different characteristic energies (keV) were used for the sample relative quantification. To quantify the EDX chemical analysis, a commercially available compact software program was used to incorporate the characteristic emitted X-ray peaks from the target sample. The error appeared owing to the geometry dynamics and the detector dead times that were deducted through the software program. Again, the EDX compositional analysis (Figure 6c) of the nylon (6/6) polymer sample showed that C, O, and Mg were the major elements having the following compositions: C—58.0%, O—23.7%, and Mg—17.0%. They measured a standard deviation (σ) of 0.8, 0.5, and 1.0, respectively. Minor elements such as Na, Al, Si, and N were present in amounts of <1% in the target sample.

### 3.5. Validation of Nylon (6/6) Functional Groups Using FTIR

**Figure 7:**
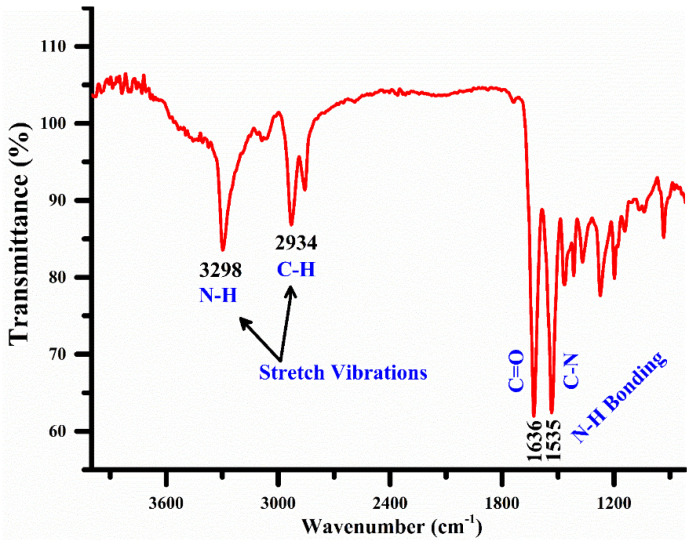
The FTIR spectral analysis of the nylon (6/6) polymer sample.

FTIR is a prevailing technique that is generally used to detect the moiety (functional groups) having structural units within the organic complexes of adsorbent samples of the comparable absorption energies and frequencies for functional molecular groups. In Figure 7, we present the FTIR spectra of the nylon (6/6) polymer sample showing medium peak bands at 3298 cm^−1^ and 2934 cm^−1^ that correspond to N-H and C-H stretching vibration, respectively, due to amino and methine groups. Similarly, strong peaks were detected at 1535 cm^−1^ (due to N-H bonding and C-N stretching) and 1636 cm^−1^ (due to C=O stretching from the carbonyl group) owing to the amide bands (I II). The peaks detected after 1500 cm^−1^ were found to be low intensity and were thus ignored. Therefore, the FTIR study demonstrated that N-H and C-N functional groups were present in our nylon (6/6) polymer sample, thus showing a good potential to construct H-bonding with the triclosan chemical molecules from hydroxyl groups.

### 3.6. XPS Analysis

**Figure 8:**
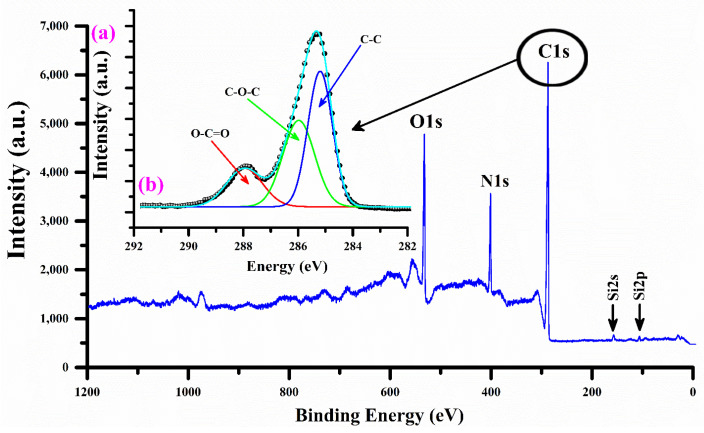
(**a**) XPS spectrum covering the binding energy region from 0 to 1200 eV and (**b**) C1s high-resolution spectra of the nylon (6/6) polymer sample.

The XPS analysis was studied with an excitation X-ray source of Al-Kα at 1486.9 eV under an ultra-high vacuum of ~10^−9^ Torr to identify the polar groups of the nylon (6/6) polymer sample [46]. The XPS spectrum of the sample having C1s high-resolution peaks (shown as an inset), as well as O1s, N1s, and Si (2s and 2p) peaks, is shown in Figure 8. The calibration process was performed using the carbon C1s peak as a reference. Various peaks of the elements C, O, N, and Si were detected at different peak energies along with their estimated peak widths, such as C1s at 285.0 (1.58), 285.9 (1.37), and 287.9 eV (1.69); O1s at 531.3 (1.7), 532.4 (1.6), and 533.8 eV (1.29); and N1s at 399.8 eV (1.72). The sensitivity factor was estimated as 1–2.5. In addition, the peak C1s at 285.0 eV were assigned to the C–C bond from CH_2_ groups. Similarly, the C1s peaks at 285.9 eV and 287.9 eV can be correlated to the following groups: amido-carbonyls [–(C=O)] and the amido-nitrogen [–C–NH(C=O)–], respectively. The composition analysis conducted by XPS on the un-doped nylon polymer sample, using an integrated peak area, further verified that carbon (>55%) and oxygen (>20%) were the predominant elements, while nitrogen (<1%) and silicon (<0.5%) were present in trace amounts.

### 3.7. Diffuse Reflectance Spectroscopy (DRS)

**Figure 9:**
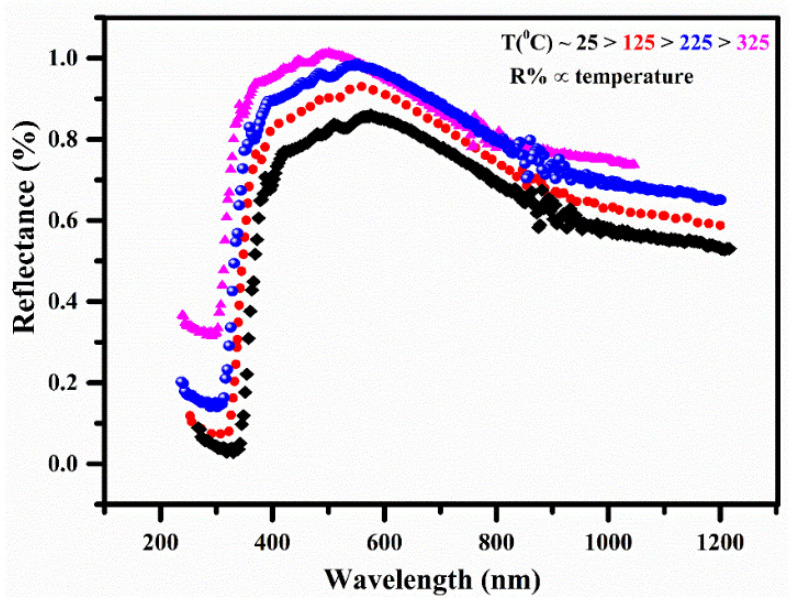
The reflectance spectra of MgO-doped nylon (6/6) polymer sample at different temperatures.

**Figure 10:**
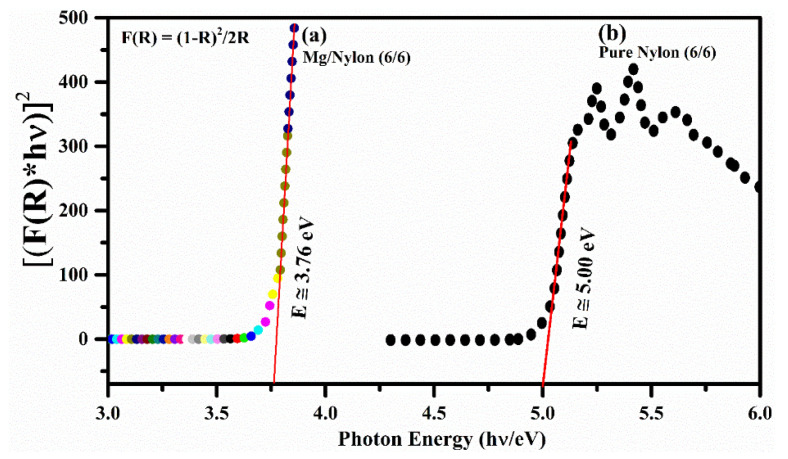
(**a**) The band gap of MgO-doped nylon (6/6) polymer sample. (**b**) Band gap of pure nylon (6/6) before doping.

The authors examined how the doped nylon polymer sample behaved in terms of thermal and reflectance properties across a temperature range of 25–325 °C. The DRS is an analytical method that is employed for investigating surface properties by providing a detailed depth profile with a high resolution, typically within the range of 10–20 nm. It effectively showcases the interaction between light and materials. Figure 9 illustrates the optical diffuse reflectance (R%) of the MgO-doped nylon (6/6) polymer sample across various temperatures (25°, 125°, 225°, and 325°) within the optical wavelength range of 200–1200 nm. The reflectance spectrum from 250–1200 nm of the polymer sample demonstrated that the R% went on growing in the UV region, declined gradually in the visible region from around 500–950 nm, and was approximately persistent in the IR region from around 950–1200 nm. The reflectance spectra of the polymer samples confirmed an enhancement of the R% by increasing the sample temperature in the UV, VIS, and IR regions, respectively. Thus, it can be associated with the increase in carrier concentration in the polymer MgO-doped sample with increasing the sample temperature, because the doped polymers generally show a low transmittance. The maximum value of the mean reflectance in the visible region around 500 nm was ~98%, while it was giving a continuously decreasing trend from 500 nm to up to 950 nm; thus, it became almost constant from 950–1200 nm. The optical band gap energy (E_g_) of the doped nylon polymer sample was estimated using the DRS reflectance spectrum following the Kubelka–Munk’s function, which is comparable to Tauc’s equation:

(4) $$F\left(R\right)=\frac{1-R^{2}}{2R}=\frac{\alpha }{s}$$

where *F*(*R*) is the Kubelka−Munk’s function related to the wavelength-dependent scattering coefficient ‘*s*’ and the absorption coefficient ‘*α*’, which is a function of the reflectance ‘*R*’ of the target sample. Thus, after ignoring the scattering coefficient ‘*s*’ due to the wavelength dependence, the expression proposed by Tauc, Davis, and Mott for the *F*(*R*) in terms of the absorption coefficient (*α*) becomes the following

(5) $${\left(F(R)E\right)}^{\frac{1}{n}}={\left(h\nu \alpha \right)}^{\frac{1}{n}}=A\left(E-E_{g}\right),$$

where ‘*A*’ is the constant of proportionality, E=hν is the photon incident energy, $E_{g}$ is the band-gap energy, and ‘*n*’ is related to the transition of the substance. For example, *n* = ½ can be taken for the direct permissible transitions, and, similarly, *n* = 3/2 for direct forbidden transitions, *n* = 2 for indirect permissible transitions, and *n* = 3 for indirect forbidden transitions, respectively. In the present work, the value of n was taken as ‘2′ for the indirect band gap of the polymer sample, and a Kubelka–Munk’s plot was obtained between ${\left(F(R)E\right)}^{\frac{1}{n}}$ and *E* as shown in Figure 10a,b. The band-gap energy was determined as 5.0 eV for the pure nylon (6/6) polymer sample and as 3.76 eV for the MgO/Nylon (6/6) sample by substituting the value of *n* as 2 for the polymer samples through the slope intercept on the energy axis. It can be seen from Figure 9 that the band gap revealed a reduction due to doping with Mg atoms in the nylon polymer sample. The decrease in the band gap owing to MgO doping signifies the merging of magnesium into the polymer atomic chain, thereby encompassing the density of states inserted into the VIS and NIR zone of the electromagnetic spectrum, which, thus, improves conduction.

### 3.8. Prinicpal Component Analsysis

We exploited principal component analysis (PCA) of the LIBS data at different target temperatures from pure nylon (6/6) and MgO-doped nylon (6/6) polymer samples to explore the features of polymers with temperature. Performing PCA on the LIBS spectral data from pure nylon (6/6) and MgO-doped nylon (6/6) polymer samples at different temperatures serves both practical and academic purposes. The analysis is motivated by the observation that the spectral lines’ intensity values change with temperature, thus leading to distinct patterns in the data. By applying PCA, we can explore and understand the features of the polymers with temperature, and we can leverage the score data plots to classify samples with different temperature levels. This approach has practical significance, as it enables quality control by identifying temperature-dependent spectral variations that allow for the classification of samples based on their temperature profiles. Simultaneously, the study has academic importance, as it enhances our understanding of the relationship between temperature, spectral features, and polymer behavior, thereby contributing to the broader knowledge base in polymer science and materials research.

PCA is an unsupervised multi-variate algebraic technique that identifies compositional vectors within a dataset by transforming primary variables, such as LIBS spectral information, into a collection of principal components [47]. The principal components are determined as linear sequences of the initial variables that are orthogonal to each other and capture the variations present in the dataset. Significant parameters in PCA, such as eigenvectors and eigenvalues, are utilized to interpret each PC factor. The eigenvectors represent the relationships between principal components and the initial LIBS data, while eigenvalues indicate the variance of the original data to each principal component. The fundamental equation that represents PCA is exceedingly straightforward [48]:

(6) $$D_{nm}=S_{nb}{\left(L_{mb}\right)}^{T},$$

where *D_nm_* is the original LIBS spectra matrix after the PCA transformation, and *S_nb_* is the scores matrix or matrix of observation (*n* rows and *b* columns). *L_mb_* is the loadings matrix (*m* rows and *b* columns). The number of factors determines the number of columns in the *S* matrix. The notation *T* as a superscript indicates the transpose operation applied to the matrix. To find the solution for the *S* matrix, multiply the data by the inverse of the principal components, which is indicated as (*L^T^*)^−1^. Keep in mind that the principal components are unit vectors. Interestingly, by taking advantage of the fact that an orthogonal matrix of unit vectors has its transpose equal to its inverse, we can simplify the process by post-multiplying it with *L* itself. In mathematical terms, the transpose of an orthogonal matrix of unit vectors is equivalent to its inverse (*S* = *DL*). Every point in S is derived by multiplying the elements of a row in *D* with the corresponding column from L and subsequently summing up the obtained values. For the loading matrix from Equation (6), an alternative approach is to pre-multiply both sides with the pseudo-inverse of the score matrix (*L^T^* = *S*^−1^*D*). Here, *S*^−1^ represents the inverse of the scores matrix. However, it should be noted that the scores matrix does not consist of unit vectors, which means pre-multiplying does not have the same effect as multiplying by the inverse.

The PCA analysis was performed using the ioGAS™ software program. To distinguish between pure and doped nylon samples, as well as their temperature dependences, we recorded the LIBS spectral data of the doped nylon sample at various temperatures (25°, 125°, 225°, and 325°). To find the standard deviation (σ) of the LIBS data, 150 spectra for each doped nylon sample at various temperatures were recorded, and 200 spectra were recorded for the pure nylon sample registered at room temperature as reference data for the training. The spectral data were then normalized and casually split into a test set (400) and a training set (200). Before performing PCA, the LIBS spectral data underwent various pre-processing steps to enhance their quality and improve the results. These steps included wavelength calibration, baseline correction, noise reduction, spectral normalization, spectral alignment, spectral binning, and outlier removal. Through these techniques, the data were refined, noise was reduced, spectra were aligned, and outliers were eliminated, thus resulting in superior outcomes during the PCA.

**Figure 11:**
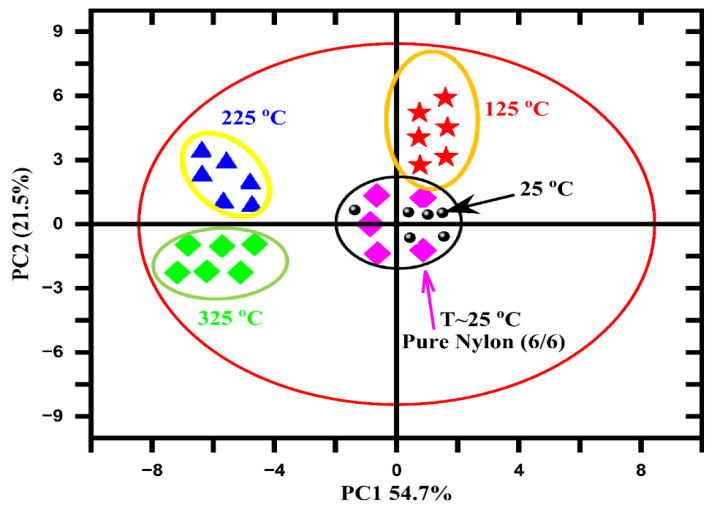
The PCA of pure and MgO-doped nylon polymer samples showing the log-transformed experimental data strategized in PC1 (54.7%) vs. PC2 (21.5%).

**Figure 12:**
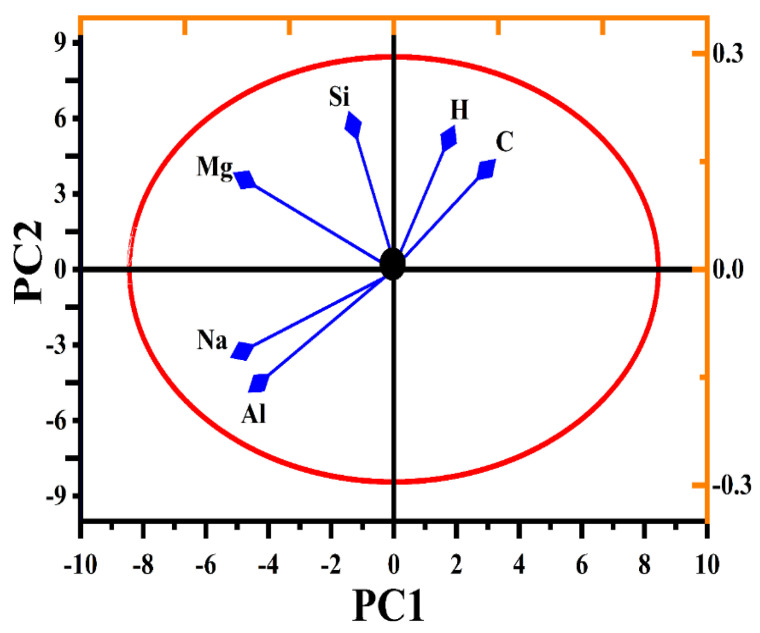
The vectors of different elements as a variable quantity in the corresponding 2D planes.

After pre-processing the LIBS data, various elements were selected that had characteristic emission lines for further analysis. A matrix containing emission lines, including their intensity (a.u.), probability amplitude factor, accuracy threshold value, and wavelength (nm), was created to explore the eigenvalues and their corresponding vectors. Two PCs had 54.7% variance for PC1 and 21.5% variance for PC2, thus showing a total of 76.2% variance of the total spectral data variance, as shown in Figure 11. The PCA of the pure and doped nylon samples was processed as having a set of 18 emission lines of six elements (H, C, Na, Mg, Al, and Si) to establish elemental variance, as shown in Figure 12. The PC1 split up the entire data of the two samples (pure and MgO/nylon), with different sample temperatures having positive loadings. Furthermore, Na and Al were the two elements having strong loadings in the samples and showed analytically negative correlations with H and C for the PC1 and PC2 (2D planes). However, C, H, Si, and Mg showed positive correlations.

Initially, we generated loading plots that illustrated the variance across different wavelengths. These plots aided us in identifying a specific set of 18 emission lines associated with six elements (H, C, Na, Mg, Al, and Si) found in the doped and five elements (H, C, Na, Al, and Si) found in the pure nylon samples. We selected these emission lines based on their significant contribution to the overall variance. Next, we compiled the data into a matrix, which included information about the elements and the number of samples. By performing the PCA analysis on this matrix, we obtained score data points. For each sample, there were six score data points derived from the six elements, which were determined by the dominant emission line out of the three lines for the doped samples as well as by the five points based on the dominant emission line out of the three emission lines for the undoped samples, as depicted in Figure 11. Consequently, while we began with 200 spectra, the final representation in the score plot consisted of six points per sample. These points originated from the specific emission lines that were selected for the analysis. However, it is important to note that we used 200 spectra in our analysis for pre-processing purposes, thus aiming to minimize statistical errors in the LIBS data.

The ellipses in Figure 11 represent confidence intervals or confidence ellipses. They provide a visual representation of the spread and clustering of the data points in the PCA score plots of LIBS data. They help assess the variance and separation of the samples in the reduced dimensional space.

In addition, PCA fails to differentiate between doped and un-doped nylon samples at room temperature (25 °C) because, while magnesium (Mg) is present in significant concentrations, other elements, including carbon (C), aluminum (Al), silicon (Si), hydrogen (H), and sodium (Na), also contribute substantially. Loading plots demonstrate that the maximum variance observed corresponded partially to C, Al, Si, Na, H, and Mg. Therefore, the clustering of samples lacked significance at room temperature in the PCA, thus indicating that the presence of Mg alone, despite its composition exceeding 15 wt.%, did not account for the distinction between the doped and un-doped nylon.

### 3.9. CF–LIBS Chemical Composition Analysis

The calculation of the chemical composition (wt.%) in CF-LIBS entails multiple steps, including the utilization of the Boltzmann equation, a well-established method, to ascertain the atomic concentration of neutral atoms [49].

(7) $$FC^{\alpha ,\gamma }=I_{ki}\frac{P^{\alpha ,\gamma }(T)}{A_{ki}g_{k}}\exp\left[\frac{E_{k}}{k_{B}T}\right]$$

Here, the partition function $P^{\gamma }(T)$ is defined as $P^{\gamma }\left(T\right)=\sum_{i}g_{i}e^{-\frac{E_{i}}{k_{B}T}}$, and $C^{\gamma }$ represents the concentration of the neutral atom. The factor *F*, which remains constant to ensure consistent efficiency of the spectral system and is associated with the ablated mass, can be determined by normalizing the concentrations of the elements found in the sample. Within the equation, $I_{ki}$ represents the integrated transition line intensity, $g_{k}$ denotes the statistical weight, $A_{ki}(s^{-1}$) represents the transition probability from (*k*) to (*i*), *E_k_* (eV) denotes the energy of the upper level, *T* denotes the excitation temperature in (eV), and *k_B_* denotes the Boltzmann constant in eVK^−1^. All the atomic factors utilized in the analysis were obtained from the NIST database [39]. The concentrations ($C^{\gamma }$) of the neutral atoms in the sample are calculated using Equation (7). To minimize the relative error, we employed the average values of the electron number density and the plasma excitation temperature during the CF-LIBS analysis.

The Saha–Boltzmann equation was utilized to determine the concentration of ionic species in the given samples. This equation establishes a connection between the concentrations of a specific element *α* in two consecutive charge states, *γ* and *γ* + 1 [47,49].

(8) $$N_{e}\frac{C^{\alpha ,\gamma +1}}{C^{\alpha ,\gamma }}=6.04\times 10^{21}\sqrt[{3}]{T_{eV}}\frac{P_{\alpha ,\gamma +1}}{P_{\alpha ,\gamma }}exp[-\frac{E_{\alpha ,\gamma }}{k_{B}T}]$$

Here, the concentration of the *γ* + 1 charge state is represented by $C^{\alpha ,\gamma +1}$, and $E_{\alpha ,\gamma }$ denotes the ionization energy of element *α* measured in electron volts (eV). $N_{e}({\mathrm{cm}}^{-3})$ refers to the electron number density, while $P_{\alpha ,\gamma +1}$ and $P_{\alpha ,\gamma }$ signify the partition functions of the upper charge state (*γ* + 1) and lower charge state (*γ*), respectively. The composition of any element in a sample is determined by combining the contributions of both the neutral state ($C^{\alpha ,\gamma }$) and the ionized state ($C^{\alpha ,\gamma +1}$).

(9) $$W_{total}^{\alpha ,\gamma }=C^{\alpha ,\gamma }+C^{\alpha ,\gamma +1}.$$

The experimental factor *F* can be approximated by normalizing the total concentration of all the elements to a value of one, as expressed by the equation $\sum W_{total}^{\alpha ,\gamma }=1$. To determine the percentage elemental compositions of different elements found in the *Saussurea simpsoniana* plant samples, the following relationship was employed.

(10) $$W^{\alpha }(\%)=\frac{C^{\alpha }}{\sum W_{total}^{\alpha ,\gamma }}\times 100.$$

In this equation, $C^{\alpha }$ represents the relative composition of each component, while $\sum W_{total}^{\alpha ,\gamma }$ represents the sum of the compositions for all the components present in the samples.

### 3.10. Comparative Study

**Figure 13:**
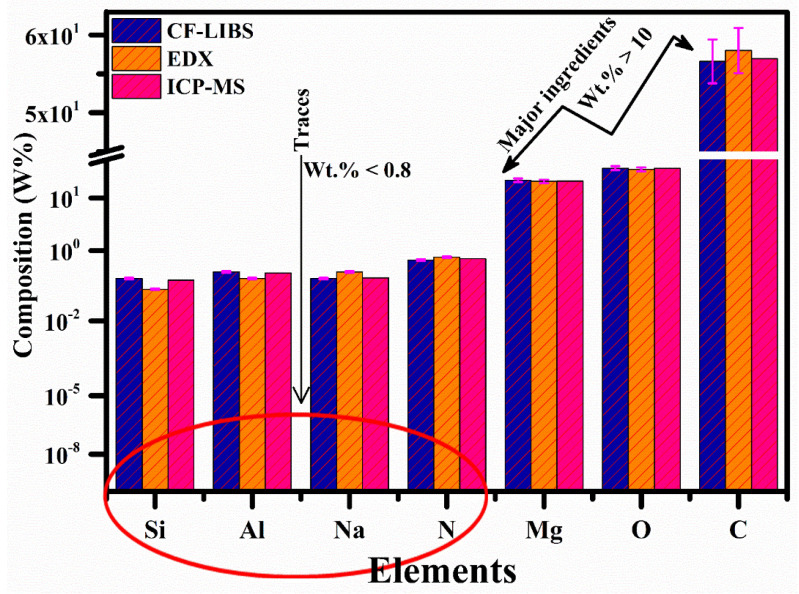
Chemical compositional (wt.%) analysis of MgO-doped nylon (6/6) polymer sample using CF-LIBS, EDX, and ICP-MS.

Through structural characterizations, including XPS, FTIR, EDX, and XRD, it was determined that carbon, oxygen, and magnesium were the primary contributing components in the nylon polymer samples. To perform a comparative analysis of the compositions, the chemical makeup of the MgO-doped nylon (6/6) polymer sample was examined utilizing CF-LIBS, ICP-MS, and EDX methodologies. Various techniques, depicted in Figure 13, can be employed for qualitative, quantitative, and multi-element analysis of the samples. However, these techniques exhibit diversity in terms of their limits of detection (LODs), precision, sensitivity, accuracy, response to matrix effects, sample handling requirements, preparation time, cost, and dead time. Figure 13 presents the outcomes obtained from CF-LIBS and EDX techniques, thus revealing that carbon (approximately 56–58 wt.%), oxygen (around 23–24 wt.%), and magnesium (about 17–18 wt.%) were the primary components in the composition. Additionally, trace amounts of silicon (below 0.3 wt.%), aluminum (below 0.4 wt.%), nitrogen (below 0.8 wt.%), and sodium (below 0.4 wt.%) were detected, with mass concentrations below 1 wt.%. The relative standard deviation error (RSDE) was determined by obtaining compositional data from the LIBS and EDX techniques and repeating the process seven times. The error bars represent the RSDE observed across the data obtained from different techniques. To validate the findings, the chemical composition obtained from CF-LIBS and EDX techniques was compared with a reference standard technique: ICP-MS. The comparative study demonstrates that our compositional results exhibited reliability with a relative error of 5% or less. Consequently, the LIBS analytical technique can be effectively utilized in the polymer industry for the chemical analysis of polymer materials.

## 4. Conclusions

This study aimed to evaluate the performance of LIBS and its compatibility with other analytical tools, including PCA, EDX, ICP-MS, XPS, DRS, and FTIR. Two nylon polymer samples, pure nylon, and MgO-doped nylon (6/6) were characterized using various techniques. Initially, the emission spectrum of the doped polymer sample was analyzed to identify elements such as C, N, Mg, Si, Al, Na, and O, as well as specific molecular emission bands such as C_2_ Swan and CN violet. The chemical concentration (%) was then estimated using the CF-LIBS technique after ensuring the LTE and optically thin plasma conditions. X-ray diffraction (XRD) analysis was conducted to examine the crystallinity of the nylon (6/6) polymer samples, while FTIR was employed to detect functional groups such as N-H, C-H, and C-N in the nylon samples. Additionally, the DRS analysis was performed to study the doping and temperature effects on the band gap and material reflectance across different sample temperatures. For chemical compositional analysis, XPS using the carbon C1s peak at 248.8 eV as a calibration reference, along with EDX analysis, was conducted. The results obtained from CF-LIBS and EDX were compared with those from ICP-MS. Furthermore, PCA was carried out using the LIBS spectral data at different target temperatures to classify the samples, thereby demonstrating the strong capability of the LIBS technique with a relative error of 5% or less.

## Figures and Tables

**Table 1:** Spectroscopic parameters of atomic silicon (Si I) are used to estimate the plasma excitation temperature.

Element	Wavelength (nm)	A_k_ (s^−1^)	g_k_	E_k_ (eV)
Silicon (I)	250.69	5.47 × 10^7^	5	4.95
	251.43	7.39 × 10^7^	3	4.93
	251.61	1.68 × 10^8^	5	4.95
	251.92	5.49 × 10^7^	3	4.93
	252.41	2.22 × 10^8^	1	4.92
	252.85	9.04 × 10^7^	3	4.93
	263.13	1.06 × 10^8^	3	6.62
	288.16	2.17 × 10^8^	3	5.08
	390.55	1.33 × 10^7^	3	5.08

## Data Availability

The data presented in this study are available on request from the corresponding author.
